# The Reduction of Adipose Tissue by Fucus Vesiculosus

**Published:** 1907-12-28

**Authors:** 


### Points in Treatment.

### THE REDUCTION OF ADIPOSE TISSUE BY FUCUS VESICULOSUS.

The one purpose for which Fucus Vesiculosus is used in medicine is for the reduction of fatness. It is always very difficult, however, to be sure how much action the drug itself exerts. Many observers have reported upon it as being of little use, but the following, though a single case, is very suggestive of its usefulness under certain conditions. It is probable that no officinal amount of fucus would reduce adiposity if the diet was not strictly limited at the same time; but in the case below, fearing that fat-reducing drugs might do harm, restricted diet was tried for a long time by itself. The summary of the case is briefly this:—

1. Rigidly restricted diet: very slow reduction in fat.

2. Rigidly restricted diet plus administration of fucus: very much more rapid reduction in weight without detriment to the general health.

### A Typical Case.

The patient was a middle-aged man of the merriest temperament imaginable. He had a rubicund face, a sparkling eye, and he was full of activity and of the kindliest fun. Unfortunately he was enormously stout, weighing nearly twenty stones, and he was getting stouter and stouter. There was no longer any room for all the fat he had in his abdomen. The result was that he had developed a huge abdominal hernia in the region of his umbilicus. The hernia did not trouble him at first, so it was left alone; but, growing continually larger as it was, it was now inconveniencing him greatly. He therefore desired to be operated upon for the removal, and if possible the cure, of this immense hernia. It was clear at once that it would be impossible to put back the contents of the hernial sac into the abdomen. There was no room for them there. They might, of course, have been amputated if they were found to be nothing more than omental fat. It was felt, however, that it would be infinitely better if the patient's adiposity could be harmlessly reduced before any operation was attempted. It was pointed out to the patient that the least harmful way of doing this was to minimise the quantity of food and drink taken daily. Like so many very fat people, the patient was by no means a great eater, but he entered thoroughly into the spirit of the matter, and rigidly adhered to a starvation diet for some weeks. The food was all weighed out for him and he was so keen that he contented himself with even less than his starvation allowance. It seemed almost cruel to give him so little to eat, but he kept up his joyous spirits all the time, and was as disappointed as his advisers that his weight went down so slowly. It was not possible for him to keep on at business while this low dietary was continued; he had to devote himself to his treatment and give up his business for the time being. Time, however, was a very important thing to him, and he could not stay away from his work indefinitely. It became urgent, therefore, that the decrease in adiposity should be accelerated if possible. It was decided to continue the starvation diet as before, but to give fucus by the mouth as well.

### The Drug.

Fucus vesiculosus is, of course, the common seaweed of the English coasts. There are two recognised preparations of it, the Extractum Fuci and the Extractum Fuci Liquidum. The former may be given in doses of from 3 to 10 grains, usually in pill form, massed with powdered liquorice or marshmallow root. The liquid extract is given as a mixture, in doses of from 1 to 2 drachms, and it was the liquid extract of fucus vesiculosus that it was decided to try here.

### How to Prescribe It.

The taste of this preparation is very nauseous, but it is somewhat masked either by liquid extract of liquorice or by syrup of lemon, the mixture being made up with chloroform water in either case. The prescription used was

| | | |
|---|---|---|
| ℞ | Extracti fuci liquidi ... ... | ʒj. |
| | Extracti glycyrrhizæ liquidi ... | ʒss. |
| | Aquam chloroformi ad ... ... | ℥j. t.d.s. |

Later 2 drachms instead of one were given three times a day.

The patient took the medicine regularly, and maintained the same diet as before. His weight immediately made rapid downward strides, and there were no ill effects. Fortunately the diminution was mainly in the abdominal adipose tissue. In three weeks' time the umbilical hernia was quite flabby and the patient laughed inordinately at the way his trousers overlapped in front. The operation was easily performed, the contents of the sac were reduced without removal, and the recovery was complete and rapid.

### Thyroid Extract.

It might be asked why thyroid extract was not used in preference to fucus. It was considered carefully but it was felt that thyroid extract, though an efficient reducer of fat, is also apt to cause dwindling of the muscles too, and a general condition which is the reverse of desirable. The heart has been much disturbed in some cases in which it has been used. It was decided that if fucus failed then thyroid extract should be resorted to, the effect being watched with extreme care. Thyroid treatment was not needed, however, the giving of liquid extract of fucus was followed by a rapid diminution in the abdominal adiposity when rigid dieting alone had previously failed to reduce it much. Whether this was merely *post hoc*, and not *propter hoc*, it is impossible to say; but it is quite certain that those who watched the above case would resort to fucus again if they happened to meet with a similar one.

